# Neurological dysfunction associated with COVID-19

**DOI:** 10.5935/0103-507X.20210042

**Published:** 2021

**Authors:** Filipa Sofia Camacho Alves da Silva, Alexei Bucur, Steeve Neves Rosado, Sílvia dos Santos Balhana, Carlos Manuel Meneses-Oliveira

**Affiliations:** 1 Internal Medicine Service, Centro Hospitalar de Setúbal - EPE - Setúbal, Portugal.; 2 Intensive Care Unit, Hospital Beatriz Ângelo - Lisboa, Portugal.

**Keywords:** SARS-CoV-2, COVID-19, Coronavirus infections, Neurological manifestations, Encephalitis, Cerebrospinal fluid, Meningoencephalitis, SARS-CoV-2, COVID-19, Infecções por coronavírus, Manifestações neurológicas, Encefalite, Líquido cefalorraquidiano, Meningoencefalite

## Abstract

COVID-19 was declared a pandemic by the World Health Organization on March 11, 2020. The clinical presentation is predominantly respiratory symptoms; however, in the current literature, several neurological manifestations associated with SARS-CoV-2 infection have been described. The authors present the clinical case of a 45-year-old man hospitalized for pneumonia with a positive test result for SARS-CoV-2, without a neurological history, who, on the sixteenth day of hospitalization, presented a sudden change in his state of consciousness accompanied by conjugated right gaze deviation and myoclonus of the face and thoracic region to the left, followed by generalized tonic-clonic seizures associated with persistent left hemiparesis. The present study highlights a positive RT-PCR test for SARS-CoV-2 in cerebrospinal fluid. The patient progressed with gradual improvement, and the outcome was favorable.

## Abstract (pt)

A COVID-19 foi declarada pandemia pela Organização Mundial de Saúde no dia 11 de março de 2020. O quadro clínico apresenta predominantemente sintomatologia respiratória, no entanto, na literatura atual, têm sido descritas diversas manifestações neurológicas associadas à infeção por SARS-CoV-2. Os autores apresentam o caso clínico de um homem de 45 anos internado por pneumonia com resultado positivo para SARS-CoV-2, sem antecedentes neurológicos, que, ao décimo sexto dia de internamento, apresentou alteração súbita do estado de consciência acompanhada de desvio conjugado do olhar para a direita e mioclonias da face e da região torácica à esquerda, seguidas de crise convulsiva tônico-clônica generalizada, associadas à hemiparesia esquerda persistente. Do estudo realizado salienta-se a existência de RT-PCR para SARS-CoV-2 no líquido cefalorraquidiano positiva. O doente apresentou evolução clínica com melhoria gradual, tendo o desfecho sido favorável.

## INTRODUCTION

On March 11, 2020, the World Health Organization (WHO) declared that the outbreak of the 2019 coronavirus (COVID-19) disease reached the pandemic level.^(1)^ The disease caused by severe acute respiratory syndrome coronavirus 2 (SARS-CoV-2) commonly manifests as a respiratory disease; however, neurological manifestations have been increasingly described.^(2)^

There are currently three potential explanatory mechanisms of how SARS-CoV-2 affects the central nervous system (CNS): transsynaptic spread of the virus, dissemination through the blood-brain barrier and immune-mediated lesions.

In the first mechanism, SARS-CoV-2 infects the olfactory epithelium, migrating via the olfactory nerve, which passes through the cribiform lamina and reaches the olfactory bulb in the CNS.^(1-5)^ Some studies have shown that in this hypothesis, the virus may present retrograde dissemination through transsynaptic transfer using endocytosis and exocytosis processes of vesicles containing the virus, with subsequent axonal transport to the bodies of neuronal cells.^(6)^ A second neurophysiopathological mechanism consists of dissemination through the blood-brain barrier, which can occur in two ways: by infection of vascular endothelial cells, which express angiotensin-2-converting enzyme receptors (ACE2), and by transport through the endothelium to CNS cells, namely, neurons and glial cells (which express ACE2 receptors), or, alternatively, by leukocyte infection (carrier of ACE2 receptors) with the ability to cross the blood-brain barrier and secondarily infect the CNS.^(2-7)^ Immune-mediated injury of the CNS seems to be secondary to the activation of innate and acquired immunity with the release of cytokines (including interleukin 6 - IL-6), chemokines, monocytes and macrophages, with consequent neurological cell damage.^(2,3)^

Vascular involvement can also generate clinical neurological presentations through SARS-CoV-2 associated cerebrovascular disease. This can be explained by two mechanisms: infection and dysfunction of endothelial cells with the release of tissue factors activating the thrombotic pathway (with consequent microangiopathy)^(2)^ and/or activation of an inflammatory pathway responsible for vasculitis or the destabilization of preexisting atherosclerotic plaques.^(2)^

Regarding neurological manifestations, cases of meningitis, encephalitis, myelitis, CNS vasculitis, disseminated acute encephalomyelitis, Guillain-Barré syndrome and stroke associated with SARS-CoV-2 infection have been described.^(2,5,8)^

The diagnosis of encephalitis presupposes the existence of brain inflammation, with or without documented pleiocytosis of the cerebrospinal fluid (CSF), with compatible alterations in imaging studies or characteristic changes on electroencephalograms. The detection of SARS-CoV-2 itself does not indicate a diagnosis of encephalitis if there is no evidence of brain inflammation or dysfunction for which there is no plausible explanation.^(2)^ Clinical cases of COVID-19 have been described in the literature that document neurological manifestations, such as changes in state of consciousness, seizures, and paresthesia, without coexisting alterations on imaging exams.^(9)^ In a literature review, eight cases of encephalitis since May 19, 2020, were described.^(2)^

The clinical case described herein by the authors aims to alert clinicians to the neurological manifestations, with special emphasis on encephalitis, that may have SARS-CoV-2 infection as a cause.

## CASE REPORT

The patient was a 45-year-old male born in Pakistan who had resided in Portugal for 7 years. He had a history of grade 3 obesity, with no other known personal history or usual pharmacological therapy.

The patient sought emergency care for fever, dry cough, dyspnea, chest pain, dysgeusia, headache and myalgia with 4 days of evolution. In the summary neurological examination at admission, there were no reported changes. In the evaluation of the respiratory system, tachypnea and pulmonary auscultation with bilateral rough vesicular murmur, without other adventitious sounds, were noted. The remainder of the objective examination showed no changes.

Of the complementary diagnostic tests performed at admission, there was a slight increase in inflammatory parameters and in arterial blood gases under an inspired oxygen fraction (FiO_2_) of 21%, with type 1 respiratory failure (Table 1), and extensive predominant bilateral, peripheral and basal opacities on chest teleradiography. After a positive reverse-transcription real-time polymerase chain reaction (RT-PCR) test for SARS-CoV-2 (nasal and oropharyngeal exudate) and negative tests for influenza A and B, *Streptococcus pneumoniae* and *Legionella pneumophila*, a diagnosis of pneumonia by SARS-CoV-2 infection was established.

**Table 1:** Complementary diagnostic tests performed during hospitalization

Complementary diagnostic test	Hospital admission	2 days after hospital admission	In the context of the generalized tonic-clonic seizure episode
Analysis			
Hemoglobin	16.1g/dL	14.3g/dL	-
Leukocytes	7.700 x 109/L	7.28 x 109/L	-
Neutrophils	78.2%	89.9%	-
Lymphocytes	17.7%	7.1%	-
C-reactive protein	16.45mg/dL	24.70mg/dL	-
Procalcitonin	1.0ng/dL	1.21ng/dL	-
Platelets	136 x 109/L	151 x 109/L	-
Creatinine	1.51mg/dL	0.91mg/dL	-
Urea	44mg/dL	40mg/dL	-
AST	84UI/L	65U/L	-
ALT	79UI/L	57U/L	-
Gamma-GT	89UI/L	78U/L	-
Alkaline phosphatase	81UI/L	76U/L	-
Total bilirubin	0.58mg/dL	0.53mg/dL	-
Direct bilirubin	0.27mg/dL	0.24mg/dL	-
Lactate dehydrogenase	613UI/L	875UI/L	-
INR	0.96	0.98	-
D-dimers	0.91mg/L	0.83mg/dL	-
Arterial blood gas analysis	Under fraction of inspired oxygen (FiO_2_) of 21%	Under high-output mask	-
pH	7.41	7.46	-
PaCO_2_	34mmHg	34mmHg	-
PaO_2_	64mmHg	85mmHg	-
HCO_3-_	21.6mmol/L	24.2mmol/L	-
Lactate	1.0mmol/L	1.1mmol/L	-
Sodium	132mmol/L	135mmol/L	-
Potassium	4.2mmol/L	4.1mmol/L	-
SatO_2_	92%	97%	-
Antigenuria			
*Legionella pneumophila*	Negative	-	-
*Streptococcus pneumoniae*	Negative	-	-
Serology			
Influenza A and B	Negative	-	-
HIV (p24 antigen + anti-HIV1/2 antibodies)	Negative	-	-
RT-PCR for SARS-CoV-2 (nasal and oropharyngeal exudate)	Positive	-	-
Lumbar puncture (cerebrospinal fluid)			
Appearance	-	-	Cloudy
Color	-	-	Colorless
Proteins	-	-	107mg/dL
Glucose	-	-	90mg/dL
Leukocytes	-	-	< 5.0 leukocytes/µL
Microbiological examination	-	-	No insulation
Alcohol-acid resistant bacteria	-	-	Negative
RT-PCR for SARS-CoV-2	-	-	Positive
Anti-Epstein Barr IgG antibodies	-	-	Positive (title: 8)
Anti-Epstein Barr IgM antibodies	-	-	Negative (title: < 2)
Anti-cytomegalovirus IgM antibodies	-	-	< 0.10
Anti-cytomegalovirus IgG antibodies	-	-	117.0UA/mL
Anti-herpes simplex 1 IgG antibodies	-	-	0.75
Anti-herpes simplex 1 IgM antibodies	-	-	< 0.10
Anti-herpes simplex 2 IgG antibodies	-	-	< 0.10
Anti-herpes simplex 2 IgM antibodies	-	-	< 0.10
VDRL	-	-	Negative
Serum analyzes			
Glucose	-	-	141mg/dL
VDRL	-	-	Negative
Electroencephalogram	-	-	No record of focal or epileptiform changes

AST - aspartate aminotransferase; ALT - alanine aminotransferase; INR - international normalized ratio; FiO_2_ - fraction of inspired oxygen; PaCO2 - partial pressure of carbon dioxide; PaO_2_ - partial pressure of oxygen; HCO_3_ - sodium bicarbonate; SatO_2_ - oxygen saturation; RT-PCR - reverse-transcription real-time polymerase chain reaction; SARS-CoV-2 - severe acute respiratory syndrome coronavirus 2; IgG - immunoglobulin G; IgM - immunoglobulin M; VDRL - venereal disease research laboratory

Over the first 48 hours, progressive worsening of fatigue, dyspnea and type 1 respiratory failure, requiring an increase in supplemental oxygen therapy, were observed. Due to the lack of improvement, noninvasive mechanical ventilation was initiated; however, due to poor adherence, high-flow oxygen therapy was initiated through a nasal cannula, without a response to therapy.

In this context, the patient was admitted to the intensive care unit (ICU), level III, where he underwent sedoanalgesia and orotracheal intubation with connection to invasive mechanical ventilation.

On the eleventh day of hospitalization, treatment with remdesivir, dexamethasone, enoxaparin and empirical antibiotic therapy with amoxicillin/clavulanic acid and azithromycin, administered on suspicion of bacterial overinfection, was continued. During this period, sustained fever was observed, with a weak response to antipyretic therapy, with improvements in inflammatory parameters after the third day of hospitalization in the ICU (Table 1).

To exclude any concomitant infectious etiology, intravenous devices were replaced, and blood cultures, cultures of the tip of the central catheter and bronchial secretions, urinalysis, urine culture and transthoracic echocardiography were performed. Among the cultures, the blood culture yielded the only positive result, i.e., *Klebsiella pneumoniae*, which is sensitive to amoxicillin/clavulanic acid, which the patient was already receiving. The summary echocardiogram did not reveal valve changes suggestive of endocarditis, but the patient presented hypokinesia of the lateral wall and left ventricular apex, as well as poor biventricular function. A slight increase in troponins (1.8ng/mL) and ST-segment depression in leads I and aVL were confirmed, suggesting the existence of acute coronary syndrome or septic cardiomyopathy.

Other noninfectious causes of febrile symptoms in the critically ill patient were excluded, including treatment with neuroleptics or altered thyroid function.

Notably, there was a need for inotropic support with dobutamine in the ventilatory weaning phase as well as noninvasive ventilatory support after orotracheal extubation, which occurred on the fifteenth day of hospitalization.

On the sixteenth day of hospitalization (nineteenth day of confirmed disease), there was an episode of altered state of consciousness, conjugated deviation of the gaze to the right and myoclonus of the face and thoracic region to the left followed by a generalized tonic-clonic seizure crisis, which ceased after midazolam therapy. The hypothesis that the seizure occurred in the context of a hypoxic-ischemic event was excluded because the patient remained normotensive, there was never a perievent or hypoxemia, serum lactate level was normal, and diuresis remained preserved. Any ionic or glycemic disorders that could explain the inaugural seizure episode were excluded.

In the post-critical period, there was an absence of eye opening, no verbal response, failure to localize to pain (coma scale of Glasgow 7), and persistent left hemiparesis grade 3 out of 5. Due to the need for airway protection, the patient was sedated, subjected to orotracheal intubation and started on anticonvulsant therapy.

In the process of diagnosis of the convulsive episode, after cranial computed tomography confirmed no changes, the patient was subjected to lumbar puncture, with turbid CSF output and mild proteinorrachia but without pleocytosis and with normal opening pressure. In the CSF, neurotropic virus and venereal disease research laboratory (VDRL), acid-alcohol resistant bacteria tests and an RT-PCR test for SARS-CoV-2 were requested, and samples were collected for culture (Table 1). Electroencephalography was performed 1 hour after propofol suspension and under fentanyl, with a single record and total duration of 13 minutes; the findings indicated no changes (Table 1).

After confirmation of a positive RT-PCR test for SARS-CoV-2 RNA in the CSF, without CSF pleiocytosis, the hypothesis of bacterial but nonviral meningitis was excluded, considering the hypothesis of encephalitis. Magnetic resonance imaging (MRI) performed on the seventeenth day of hospitalization showed multiple image artifacts associated patient movement during the procedure, suggesting the need to repeat the examination.

After discontinuation of sedoanalgesia, a change in consciousness was observed, with a Glasgow coma scale score of 14, persistent left hemiparesis with muscle strength grade 4 in 5 and an absence of involuntary movements, allowing safe orotracheal extubation in 24 hours. Given the favorable clinical outcome, brain biopsy was excluded. On the twenty-first day of hospitalization, the patient was transferred to the ward.

The patient maintained apyrexia without altered state of consciousness. No new episodes of involuntary movements were observed, and on the twenty-sixth day of hospitalization, he underwent a reassessment MRI, which revealed no pathological changes. As he maintained a favorable clinical evolution, the patient was discharged on the thirty-first day of hospitalization without antiepileptic drugs and with an appointment for follow-up with the internal medicine department.

## DISCUSSION

Several neurological manifestations associated with SARS-COV-2 infection have been recently reported in the medical literature. These studies have described the involvement of the CNS, peripheral nervous system (PNS) and musculoskeletal system. The most frequent neurological manifestation, with early onset, is headache. However, other more structural and usually later forms may appear, for example, stroke, encephalitis, meningitis, encephalomyelitis and acute myelitis. The PNS can be affected, with anosmia/hyposmia and ageusia/hypogeusia as the most frequent and early classic symptoms; however, cases of Guillain-Barré syndrome and Bell’s palsy have been reported, tending to appear later. Musculoskeletal manifestations, such as myalgia, usually earlier, and rhabdomyolysis, in a second stage, have also been described.^(2,6,10-12)^

Imaging studies may or may not show changes. Cranioencephalic computed tomography is sensitive in the identification of space-occupying lesions or hydrocephaly, and cranioencephalic MRI is especially important in the identification of white matter diseases, namely, multiple sclerosis, as well as epilepsy, particularly when affecting the temporal lobe.

In the clinical case presented, it is important to take into account that at hospital admission, the patient reported headache, without other complaints suggestive of CNS involvement. The remaining neurological manifestations appeared only on the sixteenth day of hospitalization, which is consistent with other published studies.^(6,11,12)^

An important presentation during hospitalization was the maintenance of fever even after analytical improvements in inflammatory parameters; a possible confounding factor was the isolation, in blood culture, of *K. pneumoniae*, which is sensitive to amoxicillin/clavulanic acid, medication with which the patient had already been treated.

The two MRIs performed during hospitalization, especially the second, were performed late due to the onset of symptoms, which may have decreased their sensitivity.

Considering the headache, fever and seizure and trying to frame the focal dysfunction as Todd’s palsy, in addition to a lack of pathological findings on MRI and the CSF cytochemical result, the hypothesis of viral meningitis was the most likely.

It is, however, difficult to completely exclude the existence of a fleeting inflammatory process. Despite the lack of imaging documenting changes suggestive of encephalitis, this diagnostic hypothesis should be taken into account because of the persistence of neurological dysfunction, namely, hemiparesis and slight alteration of state of consciousness, in the context of SARS-CoV-2 isolation in CSF, even in the absence of CSF pleocytosis, as described in the medical literature.^(5)^

By definition, Todd’s palsy cannot explain post-critical neurological deficits that last for more than a few hours, and in the patient described in the clinical case, the clinical presentation persisted for 4 days. The lack of vascular, meningeal or parenchymal imaging findings makes other etiologies much less likely in this context.

Although there are clinical cases described in which neurological manifestations constitute the initial clinical presentation with encephalitis,^(5)^ this clinical case, as others published,^(6,11,12)^ alerts us to the possibility of a patient with COVID-19 presenting with predominantly respiratory involvement with the clinical expression of neurological dysfunction appearing in a second stage of disease progression - in this case, 2 weeks. Sedoanalgesia, combined with mechanical ventilation, may mask part of the neurological presentation. The patient reported nonspecific headache and maintained a fever throughout hospitalization, making it difficult to accurately date the onset of neurological symptoms.

In this clinical case, there are several mechanisms that may explain CNS involvement, including direct infection by the virus, hematogenous dissemination, or even the existence of immune-mediated post-infectious disease, considering that neurological manifestations arise predominantly in the inflammatory cascade period, despite the difficulty in documenting the onset of neurological symptoms.^(13)^

## CONCLUSION

In this clinical case, the differentiation between the diagnosis of encephalitis versus viral meningoencephalitis was tenuous. However, the disclosure of this case is relevant because it alerts clinicians to the neurological manifestations, with a special emphasis on encephalitis, that may have SARS-CoV-2 infection as a cause and the possibility of a patient developing neurological conditions after initially presenting other manifestations of the disease.
